# Improving the Response of High-Grade Glioma (HGG) Cells to Temozolomide Treatment Through Combination With Imatinib

**DOI:** 10.7759/cureus.88055

**Published:** 2025-07-16

**Authors:** Georgiana-Adeline Staicu, Alexandra Costachi, Stefana Oana Popescu, Stefan-Alexandru Artene, Daniela Elise Tache, Edmond Nicolae Barcan, Andreea Denisa Hodorog, Anica Dricu

**Affiliations:** 1 Department of Biochemistry, Faculty of Medicine, University of Medicine and Pharmacy of Craiova, Craiova, ROU; 2 Department of Pharmacology, Faculty of Pharmacy, University of Medicine and Pharmacy of Craiova, Craiova, ROU; 3 Department of Obstetrics and Gynecology, Clinical Hospital Mioveni, Mioveni, ROU; 4 Department of Biochemistry, Faculty of Medicine, “Carol Davila” University of Medicine and Pharmacy, Bucharest, ROU

**Keywords:** cytotoxicity, high-grade glioma (hgg), imatinib mesylate, temozolomide, tyrosine kinase inhibitor (tki)

## Abstract

Background: High-grade gliomas (HGGs), the most common adult brain tumors, have always posed a dreaded prognosis to patients and difficulties to medical professionals due to their high heterogeneity and resistance to treatment. In terms of standard of care, maximal surgical resection along with temozolomide (TMZ) and radiotherapy (RT) has remained the current first-line treatment for HGG for decades. Surgical resection has experienced improvements due to neuronavigation and novel imaging sequences, which facilitate a more precise tumor removal while decreasing morbidity. TMZ remains the only approved first-line therapy with limited efficiency, in part because of the developing resistance and side effects. One of the characteristics of HGGs is the presence of multiple receptor tyrosine kinase (RTK) pathways that are aberrantly activated; thus, resistance to treatment as well as uncontrolled proliferation might be attributed in part to these pathways. Our current study aims to evaluate the potential cytotoxic effects of an alkylating cytostatic, TMZ, in combination with a multitarget tyrosine kinase inhibitor (TKI), namely imatinib mesylate (IMT), in comparison to their effects in monotherapy in an in vitro environment.

Methods: HGG cells growing in Roswell Park Memorial Institute Medium (RPMI) standard medium were treated with TMZ and IMT in both monotherapy and combined therapy, and the in vitrocytotoxicity was analyzed by 3-(4,5-dimethylthiazol-2-yl)-2,5-diphenyltetrazolium bromide (MTT) assay. Drug interaction (I) was classified by the multiplicative method.

Results: In the in vitro conditions, each drug exhibited cytotoxic effects in monotherapy in a dose-dependent manner. TMZ and IMT combined regimens were synergistic in 31.9%, additive in 31.9%, and subadditive in 36.1% of the combinations.

Conclusions: TMZ and IMT demonstrated cytotoxic effects in both monotherapy and combination therapy. Dual therapy yielded predominantly synergistic and additive effects, with subadditive results in approximately one-third of the combinations. Overall, combination therapy produced a better cytotoxic behavior than its individual effects in an in vitro setting.

## Introduction

Current central nervous system (CNS) WHO classification grades brain tumors as follows: astrocytoma, IDH-mutant-grades 2, 3, or 4; oligodendroglioma, IDH-mutant, and 1p/19q-codeleted-grades 2 or 3; and glioblastoma, IDH-wildtype-grade 4 [1, 2]. The shared characteristics of high-grade gliomas (HGGs) are the dismal prognosis and the high incidence; thus, intensive research in the field of neuro-oncology has been employed throughout the years. Despite remarkable advances in the understanding of molecular pathways, genetic alterations, and gliomagenesis, the cornerstone when it comes to treatment remains maximal surgical resection, radiotherapy (RT), and concurrent/adjuvant chemotherapy [3]. High-grade gliomas (HGGs) present intense cell pool heterogeneity along with a subpopulation of glioma stem cells that increase the adaptability to RT and chemotherapy, rendering more aggressive, chemo-/radioresistant cells [4].

Over the years, diagnosis and surgical management of brain tumors have benefited from improvements such as utilizing neuronavigation and intraoperative magnetic resonance imaging (MRI) to aid in the minimization of residual tumor [5]. MRI sequences, dynamic susceptibility contrast (DSC) perfusion MRI, and O-(2-[18F]fluoroethyl)-L-tyrosine positron emission tomography (FET-PET), used for either preoperative planning or for distinguishing between recurrence and pseudoprogression, are other examples of recent developments in the diagnosis, follow-up, and neurosurgical management of HGG [6]. Improvement in overall survival (OS) has been noticed with the help of multimodal imaging techniques [3]. DNA alkylating agent temozolomide (TMZ), commercially known as Temodal or Temodar, has remained the first-line agent for treating HGG for over 20 years, being one of the few chemotherapeutic agents capable of crossing the blood-brain barrier (BBB) [7]. TMZ's efficiency is highly dependent on the O6-methylguanine-DNA methyltransferase (MGMT) methylation status, conferring an increased median survival of five months in those cases. Nonetheless, chemotherapy resistance most often occurs, and tumors progress; thus, TMZ offers a modest impact on survival. Additionally, adverse reactions might hinder the administration and treatment adherence [8].

The genetic makeup of HGGs has sparked research into novel agents for the medical management of brain tumors. Since dysregulations of different growth factor signaling have been reported in HGG, several small-molecule inhibitors have been used in preclinical research [9]. One of the advantages of employing these agents is the targeted approach to cancer cells, while sparing normal cells, in comparison to traditional chemotherapy that most often targets all types of cells [10]. One of those agents, which we also focused on in the current study, is imatinib mesylate (IMT), sold under the name Gleevec or Glivec. IMT is a tyrosine kinase inhibitor (TKI), the first of this category to be FDA approved since the beginning of the century. IMT is presently the gold standard for treating chronic myeloid leukemia [11]. It also shows potential benefits in other types of tumors, such as dermatofibrosarcoma protuberans. Its main target in this tumor is the platelet-derived growth factor receptor (PDGFR) [12]. Nonetheless, IMT has important action against other tyrosine kinases (TKs) such as KIT and BCR-ABL, hence the important role in the management of hematologic malignancies and other solid tumors (gastrointestinal tumors). The PDGFR signaling pathway is potentially involved in gliomagenesis and progression of HGG in an autocrine loop fashion, with 12% of adult HGGs exhibiting mutations or amplification of platelet-derived growth factor receptor α (PDGFRA) [13]. Disruption of this pathway may result in inhibition of the tumor's growth. Moreover, it has been shown that IMT increases the radiosensitivity of HGG cells. Another potential action of IMT is the increase of drug delivery at the level of the tumor [14, 15]. Thus, in the current study, we analyze the cytotoxic effects of TMZ and IMT in combination therapy or in monotherapy in an HGG cell line.

## Materials and methods

Cell culture

This study was conducted at the Department of Biochemistry, Faculty of Medicine, University of Medicine and Pharmacy of Craiova, Craiova, Romania. The 18 HGG cell culture has been described previously in a study by Hägerstrand et al. [16]. Cells were suspended in complete Roswell Park Memorial Institute Medium (RPMI) 1640 media, containing 10% fetal calf serum (FCS) and 100 IU/mL antibiotics (penicillin-streptomycin). Cells in a cell culture flask were incubated at 37°C in a humidified incubator with 5% carbon dioxide (CO₂). When the cells reached a 70%-75% confluence, they were detached from the flask using trypsin or ethylenediaminetetraacetic Acid (EDTA) and then resuspended in fresh media and transferred to the 96-well culture plates.

Cell treatment

Stock solutions of 10 mM IMT or 100 mM TMZ were stored at -20°C±5°C, dissolved in dimethyl sulfoxide (DMSO). The stock solutions (10 mM IMT or 100 mM TMZ) used for making dilutions were stored at -20°C±5°C, dissolved in DMSO. Cells were incubated in 96-well culture plates (1-3×103 cells/well in a final volume of 200 µl/well) for 24 hours, then the cells were treated with TMZ or IMT, according to the following experimental design: the control group was treated only with diluents, the second group was treated with increasing concentrations of TMZ (1, 5, 10, 20, 25, 50, 75, and 100 μM), another group was treated with increasing concentrations of IMT (0.1, 0.3, 1, 3, 6, 10, 12, 15, and 20 μM), and four groups were treated with IMT 0.1, 0.3, 1, 3, 6, 10, 12, 15, and 20 μM in combination with 5, 10, 25, and 50 μM TMZ. The cytotoxic effects were measured at three and seven days. Every second day, the medium was discarded, the cells were washed, fresh media were added in each well, and the drugs were renewed according to the experimental design described above. The experiments were assessed independently three times.

3-(4,5-dimethylthiazol-2-yl)-2,5-diphenyltetrazolium bromide (MTT) assay

The colorimetric MTT assay was used to measure cell proliferation according to the manufacturer's instructions (Thermo Fischer Scientific, Waltham, MA). The MTT assay reduces the water-soluble, yellow tetrazolium salt MTT into an insoluble, purple formazan product. The tetrazolium MTT is reduced by metabolically active cells at 37°C; thus, the quantity of formazan that results is directly proportional to the number of viable cells. The cells were treated with 10 µl MTT solution (the MTT reagent was added to each well in a culture medium) and incubated at 37°C for four hours, then 100 µl solubilization buffer was added to each well overnight in order to dissolve the formazan crystals, and the absorbance was measured spectrophotometrically (around 590 nm). The results were expressed as the mean of optical density (O.D.).

Statistical analysis

In our study, TMZ and IMT interaction (I) was classified by the Multiplicative Method. If I1,2 = I1* I2, the effects are reported as additive; if I1,2 > I1 * I2, the effects are classified as synergistic; and if I1,2 < I1 * I2, the effects are considered antagonistic. I1 and I2 are the individual effects of two drugs, and I12 is the combined effect. This model does not include a threshold [17]. For a better understanding of synergy, consider a scenario where drug A has an x% effect and drug B has a y% effect. A multiplicative model would predict a z% effect (x*y * y/100 = z) when both A and B are present. If the observed effect is higher than the predicted effect, it suggests synergy; if the observed effect is lower, it suggests antagonism. 

Statistical analysis of the data sets was performed with the Student’s t-test with two-tailed distribution, and the results with p < 0.05 values were taken into consideration. All data are expressed as the mean and the standard deviation (SD). Experiments were performed independently three times.

## Results

TMZ-induced cytotoxicity on HGG cells 

**Figure 1:**
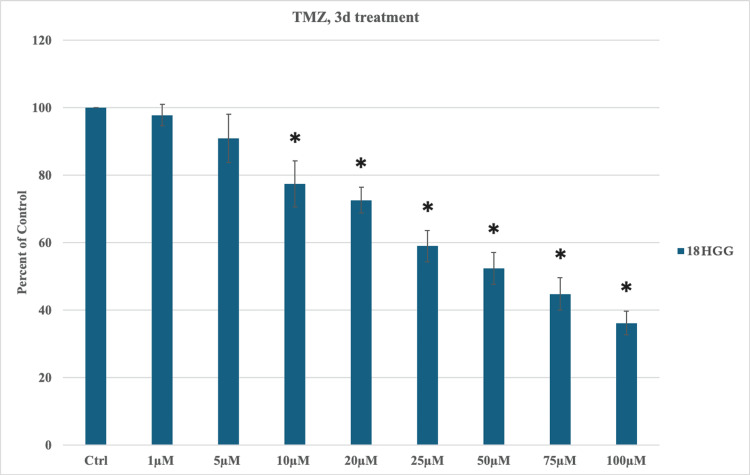
Temozolomide (TMZ)-induced cytotoxicity three days (3d) after treatment in 18 high-grade gliomas (HGG) cells. HGG cells were treated with 1, 5, 10, 20, 25, 50, 75, or 100 μM TMZ. Three days after incubation, cell viability was analyzed using the MTT assay. All data were represented as the mean of three repeated experiments ± SD (error bars). * p < 0.05 vs. control. MTT: 3-(4,5-dimethylthiazol-2-yl)-2,5-diphenyltetrazolium bromide

In the current study, we evaluated the cytotoxic effect of TMZ in monotherapy in the 18 HGG cell line at two periods of time (three and seven days), using increasing doses of TMZ (1, 5, 10, 20, 25, 50, 75, and 100 μM). During the three-day treatment, the first statistically significant cell mortality was observed at a concentration of 10 μM TMZ, with a cytotoxic ratio of 22.6%. The following increasing concentrations exhibited statistically significant cell death rates, with the highest rate being noted at 100 μM TMZ. During the three-day exposure time, cytotoxicity occurred in a dose-dependent manner, with the highest death rate (63.8%) being observed at the highest used dosage (Figure 1).

**Figure 2:**
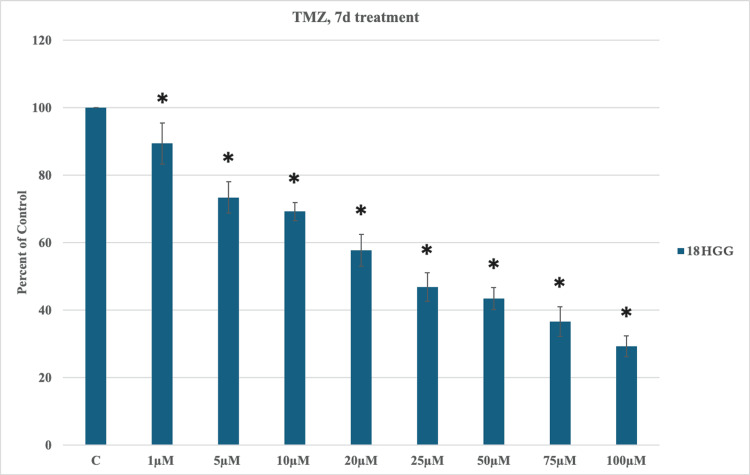
Temozolomide (TMZ)-induced cytotoxicity seven days (7d) after treatment in 18 high-grade glioma (HGG) cells. HGG cells were treated with 1, 5, 10, 20, 25, 50, 75, or 100 μM TMZ. Seven days after incubation, cell viability was analyzed using the MTT assay. All data were represented as the mean of three repeated experiments ± SD (error bars). * p < 0.05 vs. control. MTT: 3-(4,5-dimethylthiazol-2-yl)-2,5-diphenyltetrazolium bromide

TMZ-induced cytotoxicity was also evaluated at seven days. During this period of time, each dose of TMZ exhibited statistically significant cell mortality from the lowest (1 μM TMZ) to the highest (100 μM TMZ). A longer exposure time to the cytotoxic drug resulted in greater cell mortality, with the highest death rate being approximately 71% (Figure 2).

The two periods of time demonstrated a dose/time-dependent cytotoxic activity of TMZ in an in vitro cell line 18 HGG, with a continuous statistically significant decrease in cell viability (Figures 1, 2). Extending the exposure period amplified the reduction in cell viability.

IMT-induced cytotoxicity on HGG cells 

**Figure 3:**
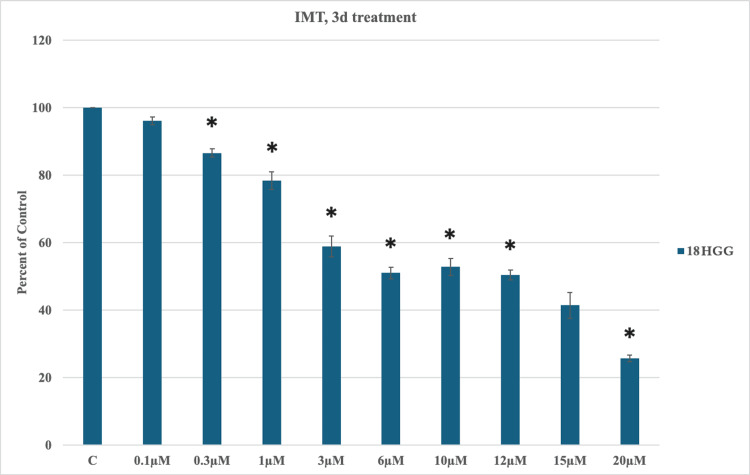
Imatinib mesylate (IMT)-induced cytotoxicity three days (3d) after treatment in 18 high-grade glioma (HGG) HGG cells were treated with 0.1, 0.3, 1, 3, 6, 10, 12, 15, or 20 μM IMT. Three days after incubation, cell viability was analyzed using the MTT assay. All data were represented as the mean of three repeated experiments ± SD (error bars). * p < 0.05 vs. control. MTT: 3-(4,5-dimethylthiazol-2-yl)-2,5-diphenyltetrazolium bromide

The effects of IMT in monotherapy on the 18 HGG cell line were assessed over a period of three and seven days at the following concentrations: 0.1, 0.3, 1, 3, 6, 10, 12, 15, or 20 μM. As illustrated in Figure 3, the cell death rate presented an increasing pattern over three days until the dosage of 10 μM, where cell viability slightly increased from 51% at 6 μM to 52% at 10 μM. Following the minimal rise in cell viability, the death rate continued its ascending trend. The highest cytotoxic effect was observed at 20 μM, resulting in a 75% reduction of cell survival.

**Figure 4:**
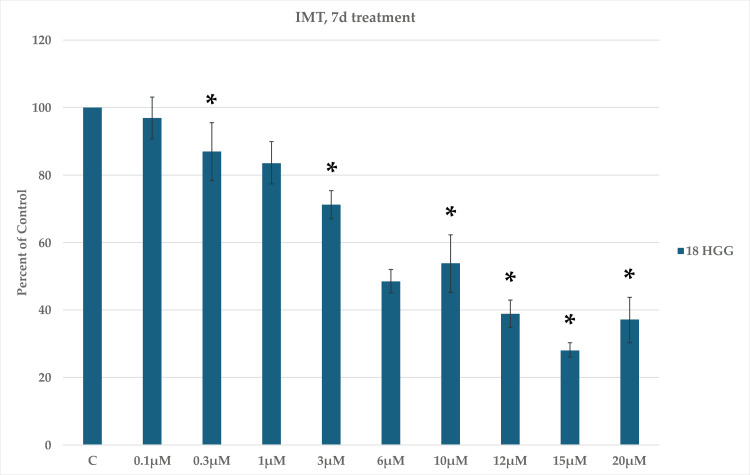
Imatinib mesylate (IMT)-induced cytotoxicity seven days (7d) after treatment in 18 high-grade glioma (HGG) cells. HGG cells were treated with 0.1, 0.3, 1, 3, 6, 10, 12, 15, or 20 μM IMT. Seven days after incubation, cell viability was analyzed using the MTT assay. All data were represented as the mean of three repeated experiments ± SD (error bars). * p < 0.05 vs. control. MTT: 3-(4,5-dimethylthiazol-2-yl)-2,5-diphenyltetrazolium bromide

Figure 4 illustrates the cytotoxic effects of IMT after increasing the exposure period to seven days under the same drug concentrations and conditions. Compared to the three-day IMT exposure time, the overall cell viability presented a steeper reduction. Nonetheless, two peaks of increased cell viability were observed at 10 μM (53%) and 20 μM (37%), respectively. In this case, the highest cytotoxicity was recorded at a concentration of 15 μM (72%).

The incubation of the 18 HGG cell line with IMT at three and seven days depicted a clear dose/time-dependent cytotoxic effect. A slight increase in cell viability at 10 and 20 μM was noted, with a better response at three days in comparison to seven days of incubation, where the highest death rate was marked (75% vs. 72%).

The effect of combined therapy on HGG cells' viability

**Figure 5:**
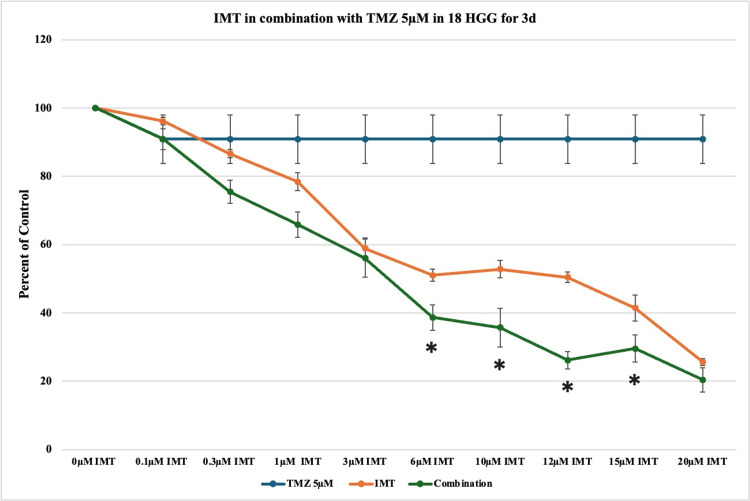
The effect of temozolomide (TMZ)-imatinib mesylate (IMT) dual therapy after three days (3d) of incubation in 18 high-grade glioma (HGG) cells. HGG cells were treated with 5 μM TMZ and 0.1, 0.3, 1, 3, 6, 10, 12, 15, or 20 μM IMT. 3 days after incubation, cell viability was analyzed using the MTT assay. All data were represented as the mean of three repeated experiments ± SD (error bars). * p < 0.05 vs. TMZ-treated cells. MTT: 3-(4,5-dimethylthiazol-2-yl)-2,5-diphenyltetrazolium bromide

Figure 5 evaluates the cytotoxic effect of TMZ (5 μM) and IMT (0.1, 0.3, 1, 3, 6, 10, 12, 15, or 20 μM) on the 18 HGG cell line over a period of three days. TMZ in monotherapy (5 μM) did not produce a statistically significant impact on cell viability (90%) across the assessed period of time. IMT in single therapy determined a progressive decrease in cell viability in a dose-dependent manner, with notable cytotoxicity at 20 μM (75%). The combination of TMZ and IMT was characterized by greater cytotoxic effects in comparison to either treatment alone. Statistically significant death rates are present at higher concentrations of IMT (≥ 6 μM), reaching a maximum of approximately 80% cytotoxicity.

**Figure 6:**
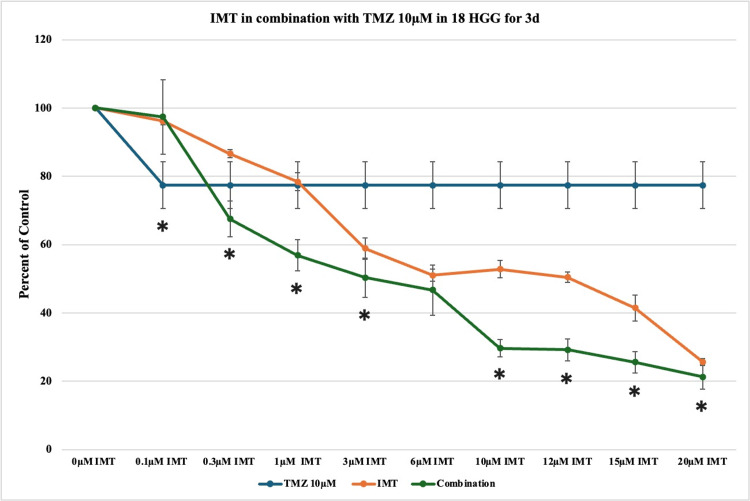
The effect of temozolomide (TMZ)-imatinib mesylate (IMT) dual therapy after three days (3d) of incubation in 18 high-grade glioma (HGG) cells. HGG cells were treated with 10 μM TMZ and 0.1, 0.3, 1, 3, 6, 10, 12, 15, or 20 μM IMT. Three days after incubation, cell viability was analyzed using the MTT assay. All data were represented as the mean of three repeated experiments ± SD (error bars). * p < 0.05 vs. TMZ-treated cells. MTT: 3-(4,5-dimethylthiazol-2-yl)-2,5-diphenyltetrazolium bromide

A concentration of 10 μM TMZ in monotherapy produced slightly better cytotoxicity (23%) in comparison to 5 μM TMZ, but with no statistically significant values. The dose-response curves of TMZ in combination with IMT exhibited statistically significant cytotoxicity with the highest registered death rate at 20 μM IMT (approximately 80%), highlighting the dose-dependent activity of both drugs (Figure 6).

**Figure 7:**
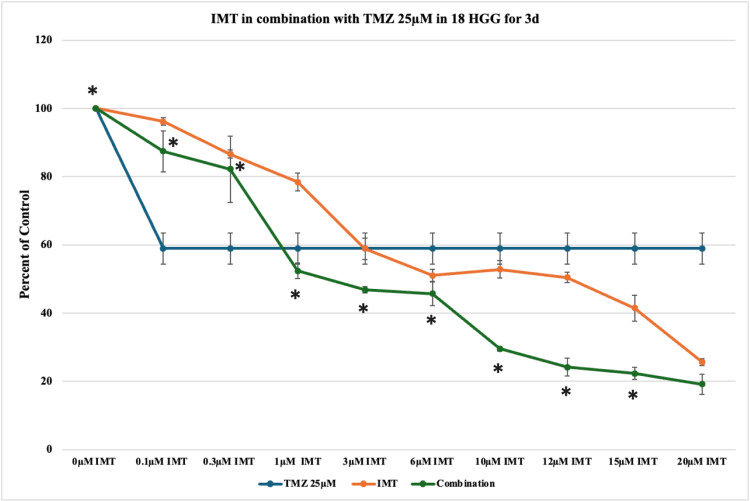
The effect of temozolomide (TMZ)-imatinib mesylate (IMT) dual therapy after three days (3d) of incubation in 18 high-grade glioma (HGG) cells. HGG cells were treated with 25 μM TMZ and 0.1, 0.3, 1, 3, 6, 10, 12, 15, or 20 μM IMT. Three days after incubation, cell viability was analyzed using the MTT assay. All data were represented as the mean of three repeated experiments ± SD (error bars). * p < 0.05 vs. TMZ-treated cells. MTT: 3-(4,5-dimethylthiazol-2-yl)-2,5-diphenyltetrazolium bromide

Increasing the concentration of TMZ to 25 μM produced a substantial cytotoxic effect, reducing cell viability to 58% over the course of three days. IMT in combination with 25 μM TMZ determined additive and synergistic effects in half of the studied concentrations, with the highest statistically significant cytotoxicity of approximately 80% at 15 μM IMT (Figure 7).

**Figure 8:**
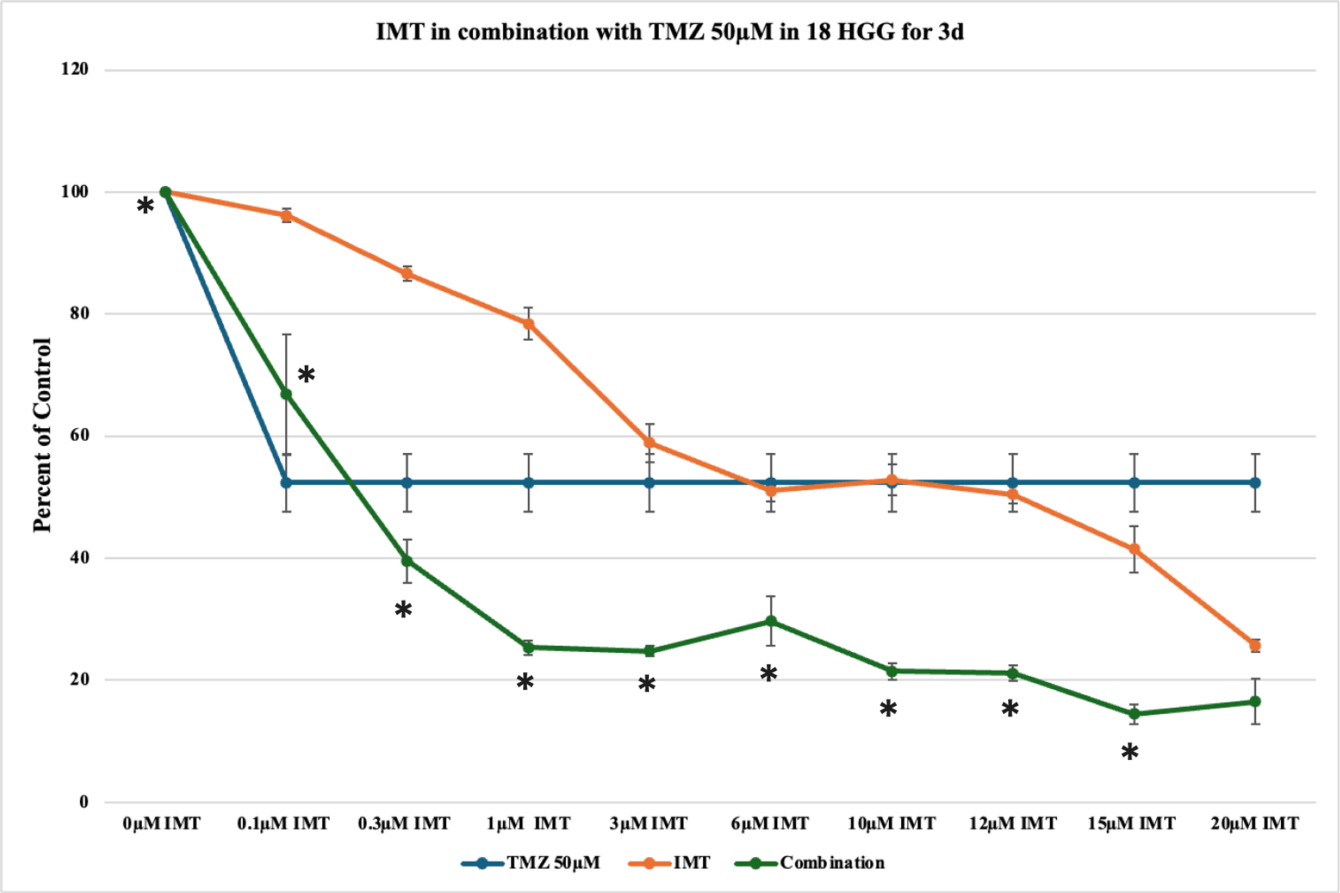
The effect of temozolomide (TMZ)-imatinib mesylate (IMT) dual therapy after three days (3d) of incubation in 18 high-grade glioma (HGG) cells. HGG cells were treated with 50 μM TMZ and 0.1, 0.3, 1, 3, 6, 10, 12, 15, or 20 μM IMT. Three days after incubation, cell viability was analyzed using the MTT assay. All data were represented as the mean of three repeated experiments ± SD (error bars). * p < 0.05 vs. TMZ-treated cells. MTT: 3-(4,5-dimethylthiazol-2-yl)-2,5-diphenyltetrazolium bromide

After increasing the concentration of TMZ in monotherapy to 50 μM, a further high cellular mortality (48%) was noted, as expected from the dose-dependent cellular response observed until now. The combination of 50 μM TMZ and IMT induced statistically significant cytotoxicity and synergistic effects, with pronounced effects at higher doses (≥10 μM). The maximal statistically significant cytotoxicity achieved was 86% at a concentration of 15 μM IMT (Figure 8).

The combination therapy of TMZ (5-50 μM) and IMT (0.1-20 μM) at three days achieved a statistically significant cytotoxicity in a dose-dependent manner. The experiments resulted in synergistic effects in 33.3% of the combinations at three days and additive effects in 41.6% of the 36 combinations, suggesting an increase in the cytotoxic activity in dual therapy.

In order to further evaluate the cytotoxic effect and the synergy, the experiment was repeated for the same doses of TMZ (5-50 μM) and IMT (0.1-20 μM) at a longer incubation period (seven days).

**Figure 9:**
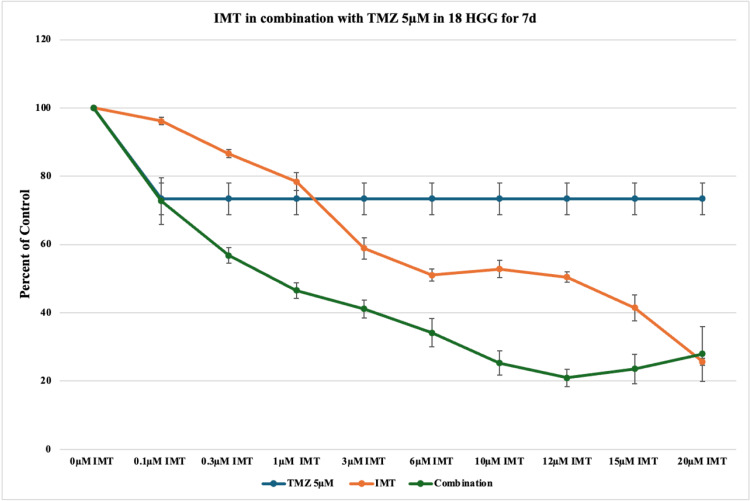
The effect of temozolomide (TMZ)-imatinib mesylate (IMT) dual therapy after seven days (7d) of incubation in 18 high-grade glioma (HGG) cells. HGG cells were treated with 5 μM TMZ and 0.1, 0.3, 1, 3, 6, 10, 12, 15, or 20 μM IMT. Seven days after incubation, cell viability was analyzed using the MTT assay. All data were represented as the mean of three repeated experiments ± SD (error bars). * p < 0.05 vs. TMZ-treated cells. MTT: 3-(4,5-dimethylthiazol-2-yl)-2,5-diphenyltetrazolium bromide

At 5 μM TMZ, cytotoxicity reached 27% in comparison to 10% at three days, resulting in a better treatment response by prolonging the exposure time. The combination of TMZ 5 μM and IMT resulted in a continuous cell death rate, reaching a maximum of 80% at 12 μM IMT (Figure 9). At these concentrations, additive and synergistic effects are mainly observed.

**Figure 10:**
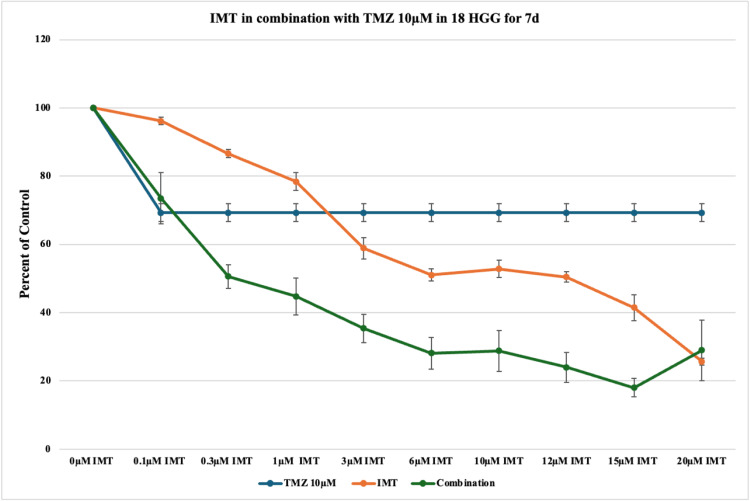
The effect of temozolomide (TMZ)-imatinib mesylate (IMT) dual therapy after seven days (7d) of incubation in 18 high-grade glioma (HGG) cells. HGG cells were treated with 10 μM TMZ and 0.1, 0.3, 1, 3, 6, 10, 12, 15, or 20 μM IMT. Seven days after incubation, cell viability was analyzed using the MTT assay. All data were represented as the mean of three repeated experiments ± SD (error bars). * p < 0.05 vs. TMZ-treated cells. MTT: 3-(4,5-dimethylthiazol-2-yl)-2,5-diphenyltetrazolium bromide

After seven days of treatment with TMZ 10 μM, a cytotoxicity of 31% was observed in comparison to 23% at three days in monotherapy. Combination therapy rendered a maximum cell death rate of 83% at seven days vs. 80% at three days (Figure 10 and Figure 6). Most of the effects were synergistic and additive in this case.

**Figure 11:**
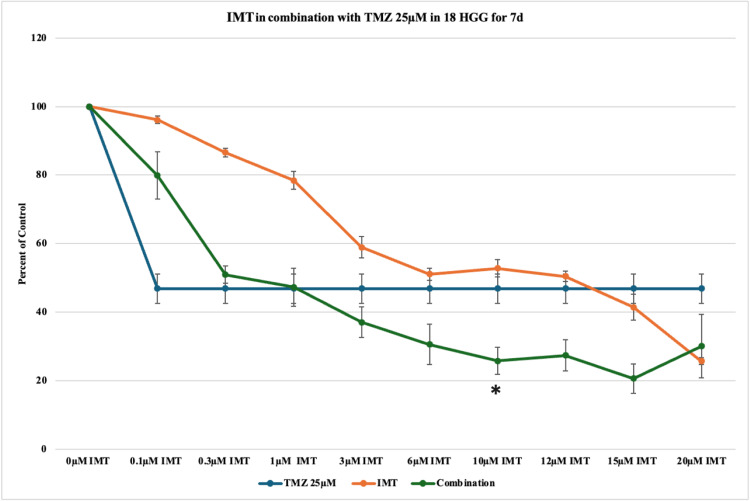
The effect of temozolomide (TMZ)-imatinib mesylate (IMT) dual therapy after seven days (7d) of incubation in 18 high-grade glioma (HGG) cells. HGG cells were treated with 25 μM TMZ and 0.1, 0.3, 1, 3, 6, 10, 12, 15, or 20 μM IMT. Seven days after incubation, cell viability was analyzed using the MTT assay. All data were represented as the mean of three repeated experiments ± SD (error bars). * p < 0.05 vs. TMZ-treated cells. MTT: 3-(4,5-dimethylthiazol-2-yl)-2,5-diphenyltetrazolium bromide

Increasing the TMZ dose (25 μM) and the exposure time produced a pronounced cytotoxic effect of up to 54% in comparison to 31% (10 μM, seven days) and 48% (25 μM, three days). The dose-response relationship of the combined therapy suggested a cytotoxicity of 80%, with greater overall cell death rates in comparison to the three-day study (Figure 11, Figure 10, and Figure 7).

**Figure 12:**
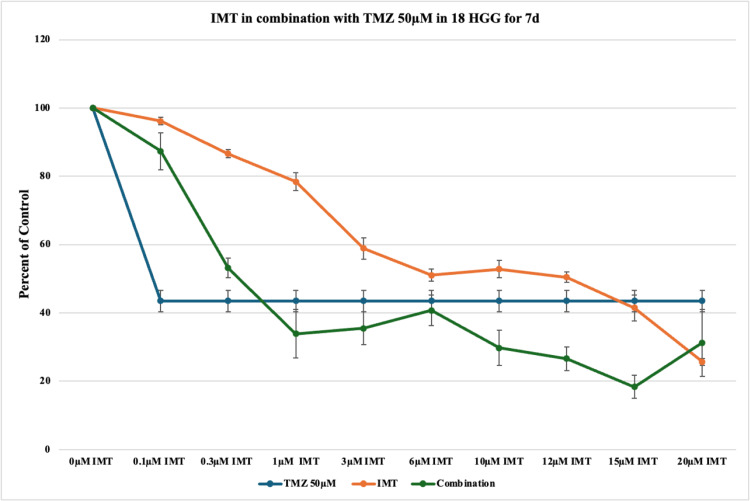
The effect of temozolomide (TMZ)-imatinib mesylate (IMT) dual therapy after seven days (7d) of incubation in 18 high-grade glioma (HGG) cells. HGG cells were treated with 50 μM TMZ and 0.1, 0.3, 1, 3, 6, 10, 12, 15, or 20 μM IMT. Seven days after incubation, cell viability was analyzed using the MTT assay. All data were represented as the mean of three repeated experiments ± SD (error bars). * p < 0.05 vs. TMZ-treated cells. MTT: 3-(4,5-dimethylthiazol-2-yl)-2,5-diphenyltetrazolium bromide

The highest dose of TMZ in monotherapy studied at the highest exposure time resulted in the greatest cytotoxic rate of 57% vs. 48% at three days. However, combination therapy did not result in better results in terms of cytotoxicity, the highest death rate being 82% in comparison to 86% achieved at three days (Figure 12 and Figure 8).

Over the seven days of incubation, TMZ in monotherapy was more effective at reducing the viability, especially at higher concentrations (lowest cell viability: 43%). The synergistic effect during the seven days was 30.5% out of 36 combinations, and the additive effect at seven days is 22.2%, resulting in an enhanced effect in dual therapy, with more pronounced cytotoxic effects.

The effects of TMZ and IMT dual therapy on HGG cells

When assessing the effects of two or more pharmacological agents, three situations may occur. The combination therapy results in subadditive effects, meaning that the experimental survival of the cancer cells exceeds the predicted survival, which is the least favorable situation, while the additive and synergistic effects are the most desirable outcomes.

In our case, the results of incubating an HGG cell line (18 HGG) with a combination of TMZ and IMT for three days yielded additive effects in 41.6% of the combinations, synergistic effects in 33.3%, and subadditive effects in 25% of 36 possible combinations (Table 1).

**Table 1:** The interaction between combined treatment in HGG cells, three days after the treatment. _*_synergistic effect; _^⁎⁎^_additive effect; _^⁎⁎⁎^_subadditive effect TMZ: temozolomide; IMT: imatinib mesylate; HGG: high-grade glioma

TMZ	IMT	Observed survival	Predicted survival
(mM)	(mM)		
5	0.1	0.9	0.9⁎⁎
	0.3	0.8	0.8⁎⁎
	1	0.7	0.7⁎⁎
	3	0.6	0.5⁎⁎⁎
	6	0.4	0.5⁎
	10	0.4	0.5⁎
	12	0.3	0.5⁎
	15	0.3	0.4⁎
	20	0.2	0.2⁎⁎
10	0.1	1	0.7⁎⁎⁎
	0.3	0.7	0.7⁎⁎
	1	0.6	0.6⁎⁎
	3	0.5	0.5⁎⁎
	6	0.5	0.4⁎⁎⁎
	10	0.3	0.4⁎
	12	0.3	0.4⁎
	15	0.3	0.3⁎⁎
	20	0.2	0.2⁎⁎
25	0.1	0.9	0.6⁎⁎⁎
	0	0.8	0.5⁎⁎⁎
	1	0.5	0.5⁎⁎
	3	0.5	0.3⁎⁎⁎
	6	0.5	0.3⁎⁎⁎
	10	0.3	0.3⁎⁎
	12	0.2	0.3⁎
	15	0.2	0.2⁎⁎
	20	0.2	0.2⁎⁎
50	0.1	0.7	0.5⁎⁎⁎
	0	0.4	0.5⁎
	1	0.3	0.4⁎
	3	0.3	0.3⁎⁎
	6	0.3	0.3⁎⁎
	10	0.2	0.3⁎
	12	0.2	0.3⁎
	15	0.1	0.2⁎
	20	0.2	0.1⁎⁎⁎

According to Table 2, seven days after TMZ and IMT combined therapy, 36 combinations resulted, with 22.2% being additive, 30.5% synergistic, and 47.2% subadditive.

**Table 2:** The interaction between combined treatment in HGG cells, seven days after the treatment. _*_synergistic effect; _^⁎⁎^_additive effect; _^⁎⁎⁎^_subadditive effect TMZ: temozolomide; IMT: imatinib mesylate; HGG: high-grade glioma

TMZ	IMT	Observed survival	Predicted survival
(mM)	(mM)		
5	0.1	0.7	0.7⁎⁎
	0.3	0.6	0.6⁎⁎
	1	0.5	0.6⁎
	3	0.4	0.4⁎⁎
	6	0.3	0.4⁎
	10	0.3	0.4⁎
	12	0.2	0.4⁎
	15	0.2	0.3⁎
	20	0.3	0.2⁎⁎⁎
10	0.1	0.7	0.7⁎⁎
	0.3	0.5	0.6⁎
	1	0.4	0.5⁎
	3	0.4	0.4⁎⁎
	6	0.3	0.4⁎
	10	0.3	0.4⁎
	12	0.2	0.3⁎
	15	0.2	0.3⁎
	20	0.3	0.2⁎⁎⁎
25	0.1	0.8	0.5⁎⁎⁎
	0	0.5	0.4⁎⁎⁎
	1	0.5	0.4⁎⁎⁎
	3	0.4	0.3⁎⁎⁎
	6	0.4	0.2⁎⁎⁎
	10	0.3	0.2⁎⁎⁎
	12	0.3	0.2⁎⁎⁎
	15	0.2	0.2⁎⁎
	20	0.3	0.1⁎⁎⁎
50	0.1	0.9	0.4⁎⁎⁎
	0	0.5	0.4⁎⁎⁎
	1	0.3	0.3⁎⁎
	3	0.4	0.3⁎⁎⁎
	6	0.4	0.2⁎⁎⁎
	10	0.3	0.2⁎⁎⁎
	12	0.3	0.2⁎⁎⁎
	15	0.2	0.2⁎⁎
	20	0.3	0.1⁎⁎⁎

The total percentage of additive effects out of 72 possible combinations at three and seven days was 31.9%; synergistic effects were present in 31.9% of the combinations, while subadditive effects were obtained in 36.1% of the combinations. The overall response to the dual therapy was positive in two-thirds of the combinations, with important synergistic and additive effects, resulting in a putative activity of the dual therapy on an HGG cell line.

## Discussion

Adjuvant therapy in HGG includes RT and the addition of an alkylating agent, both of which present limited results as chemoresistance and radioresistance develop in the HGG cell pool. Many studies have assessed the efficacy of TMZ in monotherapy on HGG cell lines sensitive to TMZ. One study evaluated the efficacy of TMZ in the U-118 cell line at 24 and 48 hours using concentrations of 10 to 500 μM, resulting in a cytotoxic effect that was dose- and time-dependent [18, 19]. In our study, TMZ yielded similar results in terms of cytotoxic effects, being most prominent at high doses and exposure times. Drug resistance usually develops through various altered signaling pathways, one of which is represented by receptor TKs (RTKs). RTKs are a major superfamily of proteins responsible for the oncogenesis and aggressiveness of HGG. Several RTKs, such as epidermal growth factor receptor (EGFR), PDGFR, and vascular endothelial growth factor receptor (VEGFR), are overexpressed and/or overactivated in HGG, further activating multiple signaling pathways involved in tumor growth, proliferation, and invasion [20, 21]. Based on this rationale, we decided to study the effects of TMZ in combination with an agent that targets multiple RTKs.

We have previously studied the impact of EGFR inhibition on treatment outcomes in two HGG cell lines, with a particular focus on the response to radiotherapy. Our results showed that individual inhibition of EGFR did not increase the radiation response in tumor cells, proving that tumoral heterogeneity and adaptability can overcome the individual targeting of one RTK [22]. PDGFR has been studied and considered to be part of the RTKs involved in the aggressiveness of HGG, with one study assessing IMT effects on subsets of HGG sensitive to PDGFR inhibition, with a reduction in cell proliferation of up to 40% [16]. IMT represents a multitarget TKI with promising results in solid tumors through PDGFR inhibition. When it comes to HGG, one study assessed the response of 26 early-passage glioma cultures to three different TKIs, namely imatinib, erlotinib, and gefitinib. The vast majority of the cultures were responders to all TKIs, while response to IMT was observed in cells with higher expressions of PDGFRA. Another trajectory for studying the effects of TKIs in HGG is their combination with chemotherapy or RT [23]. A clinical study evaluating the combination of IMT and hydroxyurea in patients with recurrent glioblastoma (GBM) showed no relevant results of combination therapy in comparison to monotherapy with hydroxyurea, while other studies reported better results of this combination in terms of overall survival [24-27]. Another study conducted by Frolov et al. demonstrated an increase in migration and motility of GBM cells treated with IMT and nilotinib. It should be mentioned that the study employed three high-passage cell lines derived from GBM [28]. Pladevall-Morera et al. have studied combination treatments of TMZ and various RTKs, including a PDGFR inhibitor, in high-grade gliomas exhibiting ATRX and PDGFR mutations, which resulted in a high sensitivity of HGG cells to these treatment combinations [29]. In one of our previous studies, we evaluated the response to RT in combination with a PDGFR inhibitor, AG1433, in two HGG cell lines. Unfortunately, radioresistance continues to be one of the major problems in treating HGG, with no direct correlation observed between the extent of receptor expression at the level of the cell membrane and response to radiotherapy, rendering surprising results [15].

Although IMT represents an efficient treatment choice in other solid tumors such as gastrointestinal stromal tumors (GIST) and in hematological malignancies, it is worth mentioning that its effects on highly heterogenic tumors like HGG are variable. In our present study, we have treated an HGG cell line with IMT and TMZ in combination and single therapy. Monotherapy yielded statistically significant cytotoxic levels in a dose- and time-dependent manner in both pharmacological agents, with the highest cell death rates registered at 71% and 75%. In dual therapy, equal percentages of synergistic and additive effects were observed, accounting for a total of 63.8%, while subadditive effects were present in 36.1% of 72 combinations. The observed cytotoxic behaviors may result from their effect on DNA integrity, such as in the case of TMZ-induced methylation, and inhibition of survival or proliferative pathways, such as PDGFR via IMT. This dual targeting may enhance cellular sensitivity to the alkylating agent and disrupt the cell cycle. Nonetheless, additional studies are needed to clarify the nature of the synergistic effects of both drugs, the molecular mechanisms of potential treatment resistance, and the impact of TKIs on various signaling pathways and genetic makeup of these highly heterogeneous brain tumors [30].

Study limitations

Our findings are obtained from one HGG cell culture (18 HGG). The in vitro conditions of one HGG cell line do not fully capture the in vivo behavior of HGGs; therefore, our results might have limitations and should be interpreted as such. Our in vitro results might not be translated to an in vivo setting, particularly in a clinical setting, since additional elements are present and generate the heterogeneity between tumors, differences in oncological patients’ outcomes, and even variability within parts of one tumor. Such elements are the microenvironment, the natural barriers present in the brain and in the tumor, and the immune response. Consequently, the true pharmacokinetics of the two drugs used in this study may not be accurately depicted, as the absence of in vivo elements may impact drug availability and metabolism.

While our study assesses the immediate cytotoxic behavior and short-term impact of single and combined IMT and TMZ therapy, further research, including animal studies, is necessary to evaluate other characteristics of HGG, such as long-term effects, systemic toxicity, resistance mechanisms, and underlying pathways through which this combination impacts viability on the cell culture, particularly in distinguishing the potential cytotoxic vs. cytostatic effects of both drugs.

## Conclusions

In this research, we investigated the in vitro cytotoxic behavior of IMT and TMZ on the HGG cell culture, 18 HGG. Proliferation and cytotoxic behavior were tested via combination index and MTT assay for both single and combination therapy, using increasing doses and incubation times. In summary, statistically significant cytotoxic levels were obtained in monotherapy in a dose- and time-dependent manner. In combination therapy, equal percentages of synergistic and additive effects were obtained, while subadditive effects were present in almost one-third of the total combinations, rendering a moderate overall effect regarding the cytotoxic behavior. Nonetheless, the complexity and heterogeneity of HGG impose the need for further research, including animal models, in order to evaluate additional factors, such as natural barriers and the immune system, and to better characterize cellular pathways that may bypass the effects of IMT in this highly heterogeneous and complex tumor.
